# Clustering Breast Cancer Patients Based on Their Treatment Courses Using German Cancer Registry Data

**DOI:** 10.1055/a-2753-9631

**Published:** 2025-12-10

**Authors:** Kolja Blohm, David Korfkamp, Florian Oesterling, Klaas Dählmann, Stefanie Schulze, Andreas Hein

**Affiliations:** 1 Research & Development Division Health, OFFIS – Institute for Information Technology, Oldenburg, Germany; 2 Cancer Registry of North Rhine-Westphalia, Bochum, North Rhine-Westphalia, Germany; 3 Division of Assistance Systems and Medical Device Technology, University of Oldenburg, Oldenburg, Germany

**Keywords:** breast neoplasms, cancer registries, cluster analysis, machine learning, treatment courses

## Abstract

**Background:**

Cancer registries collect extensive data on cancer patients, including diagnoses, treatments, and disease progression. These data offer valuable insights into cancer care, but it is challenging to analyze due to its complexity. Machine learning techniques, particularly clustering, enable the exploration of treatment data to uncover previously unknown patterns and relationships.

**Objectives:**

This work aimed to develop a method for clustering breast cancer patients in cancer registries based on their treatment courses, to demonstrate the usefulness of clustering for gaining insights, improving data quality, and identifying clinically relevant patterns.

**Methods:**

We developed a similarity measure adapted from the Levenshtein distance to compare treatment courses, incorporating cancer diagnosis, surgeries, radiotherapies, and systemic therapies. The method was evaluated on 17,822 breast cancer cases diagnosed in 2019 from the cancer registry of North Rhine-Westphalia. Evaluation involved two stages: first, domain experts reviewed the clustering results to assess clinical relevance and interpretability. Second, an intercluster survival analysis was performed to identify clinically relevant differences between treatment patterns.

**Results:**

Expert evaluations confirmed that clustering produced clinically plausible groups while also uncovering unexpected treatment patterns and potential data inconsistencies. The survival analysis showed differences in survival between clusters in both prognostically favorable and unfavorable subgroups. These results demonstrate that treatment-course clustering can identify patient groups with differing survival outcomes. However, registry data incompleteness and unmeasured confounders may influence these findings.

**Conclusion:**

Clustering treatment courses in cancer registries can reveal data quality issues, distinguish groups with different prognostic profiles, and support exploratory analyses of treatment patterns. While these findings are not intended to guide clinical decision making or evaluate treatment effectiveness, they can help generate hypotheses, identify unexpected care pathways, and support quality monitoring within cancer registries. Future work should focus on improving treatment data completeness, incorporating additional clinical variables, and refining clustering methods for broader applicability.

## Introduction

In Germany, regional cancer registries are responsible for the collection, processing, and analysis of cancer patient data. 1 Beyond basic demographic and diagnostic information, clinical cancer registries gather detailed data on disease progression and treatments administered to patients, such as surgeries or radiotherapy. Data on the course of treatment hold considerable potential for analysis. For instance, it can reveal factors influencing treatment outcomes, which in turn can improve our understanding of cancer care and treatment. However, the inherent complexity of treatment course data presents major challenges for analysis.

Traditional analyses of treatment course data often rely on predefined factors, such as adherence to clinical guidelines. While valuable, this approach often limits the exploration of broader, unanticipated patterns. Machine learning techniques, on the other hand, allow for more exploratory analyses. Among these, clustering methods can be used to automatically group patients into cohorts based on similarities and differences in their treatment courses. This is done with the goal of gaining new insights from the data, such as identifying unusual or unexpected clusters that may point to issues with data quality or highlight uncommon treatment patterns. These findings can prompt further investigation and discussion with domain experts.

The objective of our work was to develop a method for clustering breast cancer patients in cancer registries based on their treatment courses and to show that clustering can be a useful tool for cancer registries to gain new insights into their data. For this purpose, we developed a similarity measure adapted from the Levenshtein distance, 2 3 to assess treatment course similarity, incorporating a patient's cancer diagnosis, surgeries, radiotherapies, and systemic therapies. The method was evaluated using a dataset from the cancer registry of North Rhine-Westphalia (LKR.NRW), comprising 17,822 breast cancer cases diagnosed in 2019. The evaluation process included two stages. In the first stage, domain experts assessed the clinical relevance and interpretability of the clusters by examining results at three levels of granularity, coarse-grained, medium-grained, and fine-grained, facilitating the discussion of clustering results at different levels of detail. In the second stage, survival probabilities were compared across clusters to explore clinically relevant differences between treatment patterns. Our findings illustrate the potential of clustering to improve data quality, identify clinically relevant groups, and generate hypotheses for further research.

## Methods

### Data Source

In Germany, physicians, dentists, and medical institutions involved in the diagnosis or treatment of cancer are legally required to report the diagnosis and treatment of cancer cases to the relevant cancer registries. 1 The standardized reporting format in Germany is the oBDS (German: onkologischer Basisdatensatz, formerly ADT/GEKID Basisdatensatz), which defines the entities to be reported, their attributes, as well as the reporting format as an XML schema. The oBDS organizes related information into 25 groups, including tumor characteristics, treatments (e.g., surgeries, radiotherapy, systemic therapies), side effects, and disease progression. 4

For each patient, cancer registries receive multiple oBDS reports from various sources, such as general practitioners, specialists, hospitals, and pathologists. These reports may be submitted at different stages, including initial diagnosis, treatment, follow-up, or death. As a result, the registry often receives both complementary and conflicting information for the same case. To create a coherent and analyzable record, registries consolidate these inputs into a curated dataset known as the best-of-dataset, which includes validated information on both cancer diagnosis and treatment events. This dataset serves as the foundation for cancer data analysis and research. 1

To resolve different or conflicting information and compile the most accurate representation of each patient's data, registries apply a set of rules to merge and validate the data. These rules consider factors such as accuracy, plausibility, source reliability, and timeliness. For example, if tumor location is reported with varying specificity (e.g., “breast” vs. “upper outer quadrant of the breast”), the more detailed entry is retained. 1

Prioritization rules are also used to resolve conflicting data. In the case of systemic therapies, for example, multiple reports may differ in the stated intent (e.g., curative vs. palliative); here, curative intent is typically prioritized. Likewise, pathological staging is generally favored over clinical staging due to its higher diagnostic precision.

Missing values are often addressed by aggregating information from multiple reports or by deriving values from related fields. For example, inferring Union for International Cancer Control (UICC) stage from TNM classification or deducing tumor topography from ICD diagnosis codes.

Various such rules are documented in the German cancer registration manual 1 and are continuously refined by individual cancer registries as well as in collaborative working groups to promote consistency across registries. Routine plausibility checks and registry-specific validation mechanisms further enhance data quality.

Despite these efforts, data quality remains a persistent challenge. Missing values are common, and not all problems can be resolved. Moreover, some data quality issues only become apparent during analysis, prompting the development of new validation and preprocessing rules. While automated checks improve data quality, they do not detect every problem, and registries may remain unaware of certain systemic issues. Nevertheless, the best-of-datasets from German cancer registries provide a structured and comparatively reliable basis for data analysis.

The data for this work were obtained from the best-of-dataset from the LKR.NRW. The selected cohort consists of 17,822 breast cancer cases diagnosed in 2019. Table 1 provides an overview of the cohort's demographic and clinical characteristics, including age at diagnosis, tumor staging, and reported treatment modalities. Clustering was performed on diagnosis and treatment events (surgeries, radiotherapy, systemic therapies). In total, the dataset contained 47,535 events. The choice of 2019 as the diagnosis year ensures that sufficient time has passed for subsequent treatments to be administered and comprehensively reported, providing a more complete and reliable basis for analyzing treatment courses.

**Table 1:** Overview of the cohort's demographic and clinical characteristics

	Breast cancer cases *n* = 17,822 (%)
Age (y)	
Median (range)	64 (20–105)
<50	8,495 (47.7)
50–69	2,756 (15.5)
>69	6,571 (36.9)
Sex	
Male	168 (0.9)
Female	17,653 (99.1)
Other/unknown	1 (0)
Primary site	
C50.0 (nipple)	240 (1.3)
C50.1 (central portion)	1,048 (5.9)
C50.2 (upper-inner quadrant)	2,070 (11.6)
C50.3 (lower-inner quadrant)	1,105 (6.2)
C50.4 (upper-outer quadrant)	6,660 (37.4)
C50.5 (lower-outer quadrant)	1,558 (8.7)
C50.6 (axillary tail)	41 (0.2)
C50.8 (overlapping)	2,355 (13.2)
C50.9 (not otherwise specified)	2,745 (15.4)
Histological subtype	
Medullary carcinoma	54 (0.3)
Paget's disease of the breast	38 (0.2)
Inflammatory carcinoma	11 (0.1)
Tubular adenocarcinoma	131 (0.7)
Mucinous carcinoma	251 (1.4)
Invasive ductal carcinoma	11,957 (67.1)
Other histologies	5,380 (30.2)
Histopathological grading	
G1	1,878 (10.5)
G2	9,121 (51.2)
G3	5,116 (28.7)
Grading unknown	1,707 (9.6)
UICC stage	
Stage 0	189 (1.1)
Stage I	5,179 (29.1)
Stage II	4,416 (24.8)
Stage III	933 (5.2)
Stage IV	906 (5.1)
Stage unknown	6,199 (34.8)
Surgery	
Breast-conserving surgery (BCS)	7,829 (43.9)
Mastectomy	3,233 (18.1)
Other breast surgery	10 (0.1)
Other surgeries	325 (1.8)
None/unknown	6,425 (36.1)
Systemic therapy	
Yes	6,150 (34.5)
No/unknown	11,672 (65.5)
Radiotherapy	
Yes	5,552 (31.2)
No/unknown	12,270 (68.8)

The dataset represents curated data with generally high quality. A strength of German cancer registry data is a high proportion of histologically verified diagnoses (generally above 85% due to external quality control requirements). There are known data quality issues, such as missing values, especially in stage information, where missingness often exceeds 30% of cases. Cases with missing information were deliberately retained and missing values were treated as separate categories in the computation of distance measures. This approach allows us to evaluate whether the clustering method performs robustly on a dataset that is representative of typical registry data and to explore whether clusters associated with data quality issues can be identified. Furthermore, a particular trait of the therapy data is that, in contrast to, for example, stage information, it is not possible to distinguish whether a therapy was simply not documented or whether no therapy actually took place. Within the scope of this study, it was not possible to validate the patients' medical histories using external information.

### Comparing Breast Cancer Patients Based on Their Course of Treatment

In this section, we describe our approach for comparing the treatment courses of breast cancer patients, with the goal of assessing their similarity. The approach consists of two main steps: first, determining the similarity of individual treatment events based on selected attributes, and second, aggregating these event-level similarities to calculate the overall similarity between treatment courses using a modified Levenshtein distance. 2 3

We begin by describing the first step, the comparison of individual treatment events: A treatment course is represented as a sequence of events, encompassing various types. For this analysis, we focus on four primary event types: the initial diagnosis event, surgery events, radiotherapy events, and systemic therapy events.

Each event type has a complex, multidimensional schema that includes numeric, categorical, and Boolean attributes, as well as nested objects and lists. To compare two events, we calculate the distance between their individual attributes. These attribute-level distances are normalized to a scale of 0 to 1, ensuring comparability across different types of attributes.

Two types of attribute values are treated differently in distance calculations: missing values and unrelated values.

- Missing values arise from incomplete documentation, nonreporting, or explicit coding as unknown or not ascertainable.
- Unrelated values are those that fall outside the set of values considered meaningful for comparison in the context of breast cancer. These are defined based on expert-driven criteria for each attribute. For example, in the case of histology, only six specific codes are used to compute distance; all other histology codes are treated as unrelated. Similarly, surgeries that cannot be clearly assigned to one of three breast surgery categories are treated as unrelated.

Neither missing nor unrelated values are removed from the dataset. Instead, they are retained and assigned the maximum possible distance from themselves and all other values. This prevents them from artificially increasing the similarity between treatment courses. This treatment applies to all attributes, even if not explicitly mentioned in each case.

To determine the similarity of two events, the weighted average of their attribute distances is calculated. This results in a similarity score ranging from 0 (identical events) to 1 (maximally different events). Events of different types, such as surgery and radiotherapy, are inherently incomparable and are assigned the maximum distance of 1.

With event-level similarity scores established, the next step is to compare entire treatment courses. This is achieved using a modified Levenshtein distance, adapted for the comparison of treatment sequences. The Levenshtein distance is a well-known similarity measure that calculates the minimum number of operations, i.e., insertions, deletions, or substitutions, required to transform one sequence into another. For treatment courses, the insertion and deletion costs are fixed at 1, whereas the substitution cost is defined by the event-level similarity score. To normalize the overall distance, it is divided by the maximum length of the two sequences being compared, ensuring that the results are comparable regardless of sequence length.

This methodology provides a framework for assessing the similarity of treatment courses by combining detailed event-level comparisons with sequence-level aggregation. The following subsections detail the attributes and distance functions used for individual treatment events.

#### Diagnosis

To determine the similarity of two diagnosis events, we use the UICC stage, the Eastern Cooperative Oncology Group (ECOG) performance score, age at diagnosis, histology, and grading. The total distance between two diagnoses is calculated as the weighted sum of distances, with all five attributes weighted equally.

- UICC Stage: The UICC Stage is ordinal-scaled, with values mapped to numerical values from 0 to 4, and the distance is determined as the normalized Euclidean distance:
  ○ UICC 0: value 0
  ○ UICC I: value 1
  ○ UICC II: value 2
  ○ UICC III: value 3
  ○ UICC IV: value 4
- ECOG score: Similar to the UICC stage, the ECOG score ranges from 0 (fully active) to 4 (completely disabled), and the distance is calculated as the normalized Euclidean distance:
  ○ Fully active: value 0
  ○ Some restrictions: value 1
  ○ Ambulatory: value 2
  ○ Limited self-care: value 3
  ○ Completely disabled: value 4
- Age at Diagnosis: Age is grouped into three categories based on breast cancer screening participation age, the distance is 0 if the age groups are identical and 1 if they are not:
  ○ Under the eligible age (<50)
  ○ At the eligible age (50-69)
  ○ Above the eligible age (>69)
- Histology: Six groups are considered for histology distance, the distance is 0 if the groups are identical and 1 if they are not:
  ○ 8510/3 (medullary carcinoma)
  ○ 8540/3 (Paget's disease of the breast)
  ○ 8530/3 (inflammatory carcinoma)
  ○ 8211/3 (tubular adenocarcinoma)
  ○ 8480/3 (mucinous adenocarcinoma)
  ○ 8500/3 (invasive ductal carcinoma/NST)
- Grading: The tumor grading is mapped to numerical values for calculating the normalized Euclidean distance as follows:
  ○ Malignant melanoma of the conjunctiva: value 0
  ○ Borderline: value 1
  ○ Well-differentiated: value 2
  ○ Low grade (G1 or G2): value 3
  ○ Moderately differentiated: value 4
  ○ Intermediate (G2 or G3): value 5
  ○ Poorly differentiated: value 6
  ○ High grade (G3 or G4): value 7
  ○ Undifferentiated: value 8

#### Systemic Therapy

The similarity of systemic therapies is calculated based on two factors: the temporal relationship to the primary surgery and the type of systemic therapy. The type of systemic therapy is given a higher weight of 0.7, whereas the temporal relationship to the primary surgery is weighted at 0.3.

- Temporal Relationship to Primary Surgery: The temporal relationship to primary surgery refers to the timing of the systemic therapy relative to the primary surgery, the distance is 0 if the relationships are identical and 1 if they are not:
  ○ Adjuvant (A)
  ○ Neoadjuvant (N)
  ○ Intraoperative (I)
  ○ Without relation to surgery (O)
- Systemic Therapy Types: For distance calculation we consider the following systemic therapy types: Chemotherapy (CH), hormone therapy (HO), immune/antibody therapy (IM), targeted substances (TS), chemotherapy + immune/antibody therapy, chemotherapy + targeted substances (CT), and chemotherapy + immune/antibody therapy + targeted substances (CIT), Immune/antibody therapy + targeted substances (IT), Stem cell transplantation (including bone marrow transplantation) (SC), Active Surveillance (AS), Wait and see (WS), Watchful Waiting (WW), Other (OT), and Missing (MI). The distance matrix, used for calculating the distance between systemic therapy types, was developed by domain experts from the LKR.NRW. The distance matrix is shown in Table 2 .

**Table 2:** Distance matrix used to compare systemic therapy types

Distance	CH	HO	IM	TS	CI	CT	CIT	IT	SC	AS	WS	WW	OT	MI
CH	0	0,8	0,8	0,8	0,4	0,4	0,4	0,8	0,5	0,9	1	1	1	1
HO		0	0,2	0,2	0,8	0,8	0,8	0,2	1	0,8	0,8	0,8	1	1
IM			0	0,2	0,7	0,8	0,7	0,2	1	0,8	0,8	0,8	1	1
TS				0	0,8	0,7	0,7	0,2	1	0,8	0,8	0,8	1	1
CI					0	0,2	0,1	0,8	0,5	0,9	1	1	1	1
CT						0	0,1	0,8	0,5	0,9	1	1	1	1
CIT							0	0,7	0,5	0,9	1	1	1	1
IT								0	1	0,8	0,8	0,8	1	1
SC									0	1	1	1	1	1
AS										0	0,2	0,4	1	1
WS											0	0,2	1	1
WW												0	1	1
OT													0	1
MI														1

#### Surgery

The similarity of surgeries is calculated based on the procedure codes, residual status, and complications. The weighting is set to 0.5 for procedure codes, and 0.25 each for residual status and complications.

- Procedure Codes: Each surgery has a list of procedure codes. These codes are used to categorize surgeries into one of three groups. For surgeries that cannot be unambiguously assigned to one of the three groups, the surgery type is considered missing/unrelated. The distance between surgeries assigned to the same group is 0, while the distance between surgeries assigned to different groups is 1. The categorization is as follows:
  ○ Breast-conserving surgeries (BCS) (codes starting with 5-870)
  ○ Mastectomies (codes starting with 5-872, 5-874, or 5-877)
  ○ Other breast surgeries (codes starting with 5-879)
- Residual Status: The residual status describes the tumor residues that remain after the surgery. The mapping to numerical values for calculating the normalized Euclidean distance is as follows:
  ○ R0 (no residual tumor): value 0
  ○ R1(is) (in situ residue) and R1(cy + ) (cytologic residue): value 1
  ○ R1 (microscopic residual tumor): value 2
  ○ R2 (macroscopic residual tumor): value 3
- Complications: Complications are represented as a list of categorical values. We use the Jaccard distance 5 to calculate their distance.

#### Radiotherapy

The similarity of radiotherapies is calculated based on their application types, target regions, and temporal relationship to the primary surgery. The weighting is set at 0.5 for the target region, and 0.25 each for the application type and the temporal relationship to the primary surgery.

- Application Types: A radiotherapy event comprises a series of radiations, each with specific target regions and intended applications. The application type can be one of four values. To facilitate comparison, the application types of individual radiations are aggregated into a list, and the Jaccard distance is used to calculate the distance: The application type values are as follows:
  ○ Percutaneous
  ○ Metabolic
  ○ Contact
  ○ Other
- Target Regions: The target regions of all radiations are aggregated into a single value. If at least one target region other than the breast or thorax is irradiated, the target region of the radiotherapy is treated as missing/unrelated. A distance of 0.5 applies between 'breast' and 'thorax', and a distance of 0 applies for identical values:
  ○ 'Breast', if only the breast is irradiated
  ○ 'Thorax', if the thorax (and possibly the breast as well) is irradiated
- Temporal Relationship to Primary Surgery: The distance for the temporal relationship is calculated in the same way as for systemic therapy, with a distance of 0 if the relationships are identical and 1 if they differ.

### Clustering Process

The similarity measure described in the previous section is used for a pairwise comparison of treatment courses. The resulting distance matrix is then processed using Uniform Manifold Approximation and Projection (UMAP) 6 for dimensionality reduction. There are two main reasons for applying UMAP before clustering: first, UMAP's ability to capture the topological structure of the data and increase its separability can improve clustering performance. 7 8 Second, projecting the data into a 2D Euclidean space facilitates the visualization of both the similarity of treatment courses, as computed by our similarity measures, and the subsequent clustering results.

The dimensionality reduction with UMAP can be influenced by several parameters, with *n_dimensions* , *n_neighbors* , and *min_dist* being especially important 9 : *n_dimensions* specifies the dimensionality of the reduction and is set to 2 for a reduction to 2D Euclidean space. *n_neighbors* specifies the size of the local neighborhood. Smaller values emphasize the global structure of the data, whereas larger values give more weight to the local structure around individual points. *min_dist* determines the minimum distance between points in a neighborhood. The specific selection of these parameters is specified in the respective sections of the evaluation.

While UMAP includes a seed parameter for reproducibility, minor variations across operating systems and hardware architectures have been reported due to differences in numerical precision and random number generation a . These variations can lead to slight differences in the resulting embeddings, even when the same seed is used. Although they do not produce fundamentally different structures, they can subtly influence clustering outcomes.

## Evaluation and Results

The evaluation of our approach to clustering breast cancer patients was conducted in two stages. In the initial evaluation stage, an expert review was conducted with domain experts from the LKR.NRW. The objective of this stage was to assess the clinical relevance and interpretability of the identified clusters from the perspective of potential users. In the subsequent stage, the survival probabilities of the patients were examined in an intercluster comparison. This was to address the question of whether clinically relevant differences between the clusters could be identified based on a specific clinical question.

### Clinical Relevance and Interpretability

Given our main use case for clustering data in clinical cancer registries is to help cancer registries gain new insights into their data, we first discussed clustering results with experts from the LKR.NRW. We selected and clustered a cohort consisting of 17,822 breast cancer cases diagnosed in 2019 as our data basis.

#### Evaluation Setup

To facilitate the discussion of clustering results at different levels of detail, and in the absence of a predefined meaningful number of clusters for content evaluation, we divided the number of clusters into three different ranges: coarse-grained (1–50 clusters), medium-grained (51–100 clusters), and fine-grained (101–200 clusters). A smaller number of clusters provides an initial overview, which can be explored in greater depth in subsequent steps. In agreement with the domain experts from the LKR.NRW, we decided that more than 200 clusters were too large for meaningful content analysis. For each level of detail, we selected the clustering method and parameter settings based on the Silhouette score. 10 In general, it is desirable for clustering methods to form clusters that are as homogeneous as possible within the clusters and as heterogeneous as possible between them. The Silhouette score combines the homogeneity within the clusters and the heterogeneity between the clusters into a single score, making it well suited for selecting parameter settings.

The clustering results were presented to and discussed with domain experts from the LKR.NRW. Six staff members were present for the discussion: three from the cancer registry who were already involved in the design of our clustering method and three from the clinical evaluation center. The participants were given a brief explanation of the basics necessary for understanding and interpreting the results, such as the fundamentals of clustering and an introduction into the visualizations used during the discussion. The following discussion with the experts focused on assessing the quality of the clustering results. To assess the plausibility of the clusters, various clusters from the three different levels of detail were examined in detail.

##### Choosing Clustering Algorithms and Parameters

First, the parameters of the UMAP algorithm used for all clustering procedures were determined. The important UMAP parameters were briefly described in section 2.3. The selection was based on a visual inspection of different parameter combinations. The choice of settings was mainly based on the level of detail we wanted to be able to distinguish between the clusters. This may also depend on the application. For instance, if we are interested in small differences between very specific treatment courses, a small value for *n_neighbors* should be chosen. For a broader, coarser distinction, a larger value may be appropriate. As we are not investigating a specific question but rather making a general assessment of the usability and relevance of clustering, we chose a parameter combination that we visually assessed as a balanced projection between local and global structures. We used the umap-learn Python library with *n_neighors* set to 25, *min_dist* set to 0.1, *metric* set to 'precomputed', a random seed of 42, and all other settings left to their defaults. The resulting embedding was clustered using three different clustering algorithms: agglomerative hierarchical clustering, DBSCAN, and HDBSCAN.

Hyperparameter values for the clustering algorithms were selected using a grid search based on the Silhouette score. For DBSCAN, the grid search was performed using different combinations of *eps* and *min_samples* . Similarly, for HDBSCAN, different combinations of *min_samples* and *min_cluster_size* were employed. For hierarchical clustering, the complete linkage method was utilized, and the parameter *distance_threshold* was varied. For each level of detail (1–50 clusters, 51–100 clusters, and 101–200 clusters), we selected the clustering method and parameter settings with the highest Silhouette score. Additionally, the number of outliers was limited to a maximum of 5% of the total data.

##### Visualizations

**Fig. 1:**
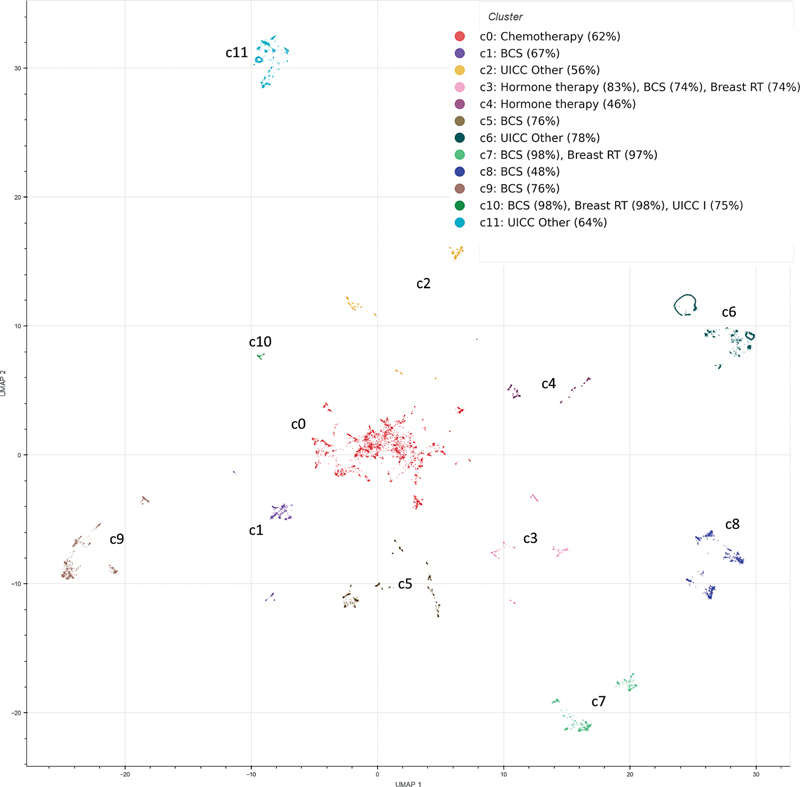
This figure shows the clustering results at the coarsest level, displaying 12 initial clusters labeled for easier identification. BCS, breast-conserving surgery; RT, radiotherapy.

The manual assessment of the cluster results is supported by various visualizations and descriptive statistics. The UMAP embeddings resulting from the similarity calculation are visualized in a scatter plot, colored according to the cluster assignment, to gain an initial impression of the cluster composition ( Fig. 1 ). The legend of the scatter plot shows a summary of the most frequent events for each cluster. Since it is not feasible to display all attributes of an event at once, each event is characterized by the attribute with the highest weight in the distance calculation. For diagnoses, where all attributes are weighted equally, the UICC attribute is used; for surgeries, the type of surgery (BCS, mastectomy, other breast surgery); for radiotherapy, the target region (breast, thorax); and for systemic therapies, the type of systemic therapy (e.g. chemotherapy, hormone therapy, etc.).

**Fig. 2:**
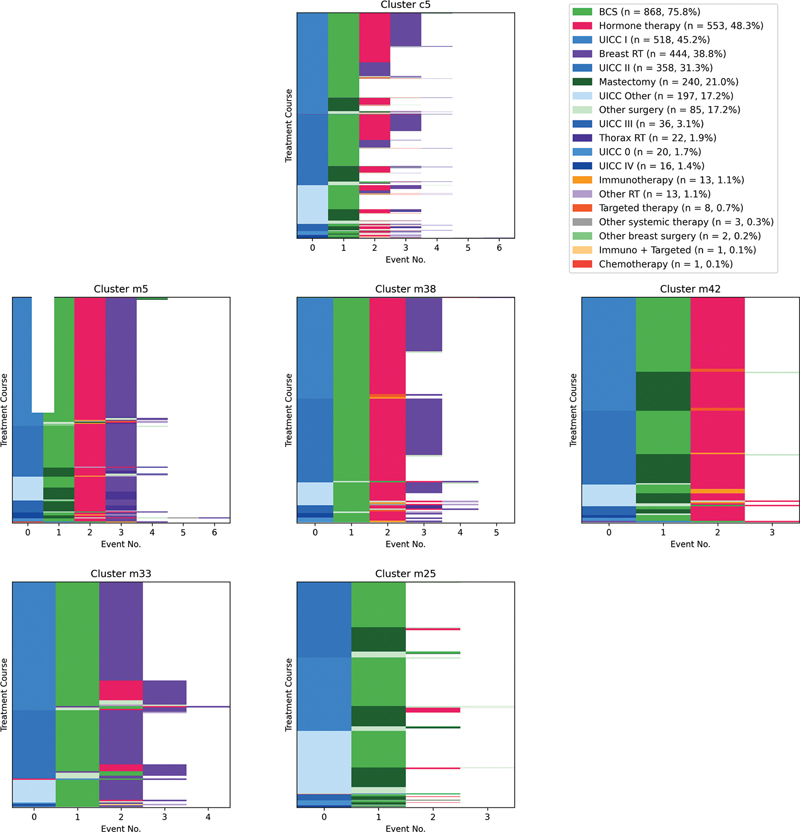
Sequence diagrams illustrating cluster c5 at the coarse-grained level and its subsequent subdivisions into five clusters at the medium-grained level. Each row in the diagrams represents a treatment course, and each cell in a row represents a treatment event, displayed by their most important attributes. Cluster c5 consists primarily of multitreatment courses, which are further divided into clusters m42, m5, m38, m33, and m25. Cluster m42 includes cases of BCS followed by hormone therapy. Cluster m5 contains cases of BCS followed by hormone therapy and additional radiotherapy. Cluster m38 consists of cases with BCS followed by hormone therapy, with about half of the cases having additional radiotherapy, differing primarily in terms of age group. Cluster m33 includes cases of BCS followed by radiotherapy. Cluster m25, containing patients younger than 50 with only one surgery, stands out as it does not consist of multiple treatments. The legend is displayed only for cluster c5, with the coloring also applicable to the other sequence diagrams. BCS, breast-conserving surgery; RT, radiotherapy.

Using tooltips and various selection methods, it is also possible to view individual treatment courses of the clusters in detail. Furthermore, each cluster is characterized by its medoid treatment course, i.e., the course that differs least from the other courses in its cluster in terms of the distance measure. In addition, both the five most similar and the most dissimilar courses to the medoid course are listed. The treatment courses assigned to a cluster are displayed in a sequence diagram (see Fig. 2 for an example). Each row in the diagram represents a treatment course, and each cell in a row represents a treatment event. Events are again represented by their most important attribute. For a detailed examination of the differences between individual clusters, the distribution of the characteristics of individual attributes was examined where necessary.

#### Results

Table 3 shows the clustering methods and parameter settings that resulted in the highest Silhouette scores, along with the minimum, maximum, and median sizes of the clusters. In all three cases, the hierarchical clustering method produced the best Silhouette scores.

**Table 3:** Clustering methods and parameters with the highest Silhouette scores

Granularity	Clustering method	# Clusters	Silhouette score	Min. cluster size	Max. cluster size	Median	25% quartile	75% quartile
Coarse-grained (1–50 clusters)	Hierarchical clustering, distance_threshold 15	12	0.68	130	4,986	1160.5	784.75	1741
Medium-grained (51–100 clusters)	Hierarchical clustering, distance_threshold 3	53	0.66	6	1,428	268	156	455
Fine-grained (101–200 clusters)	Hierarchical clustering, distance_threshold 1	174	0.61	1	455	91	48	131

Abbreviations: Max., maximum; Min., minimum.

**Fig. 3:**
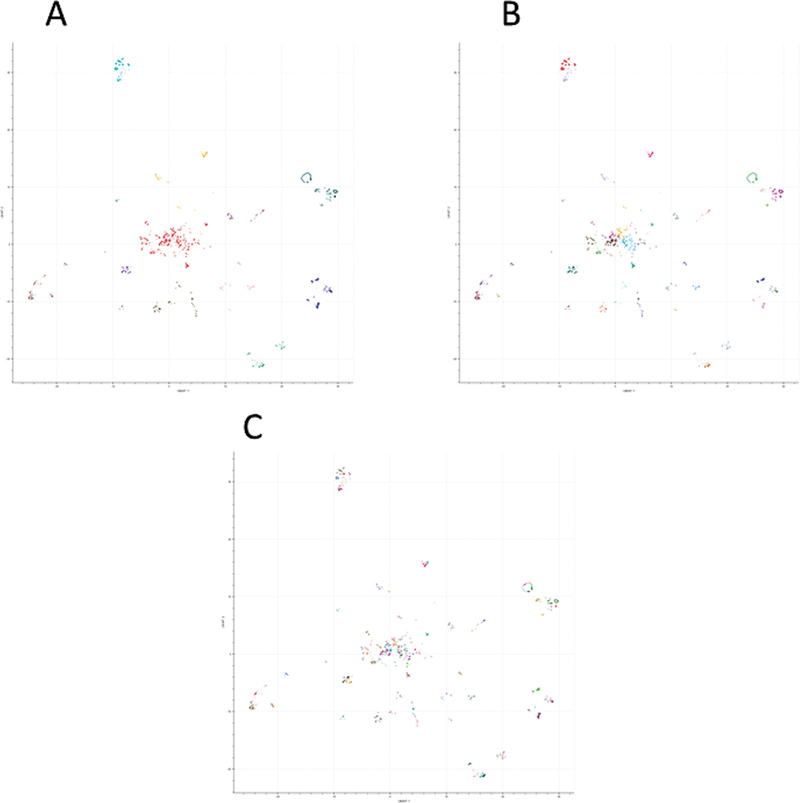
Visual representation of the coarse-grained (A), medium-grained (B), and fine-grained (C) clustering results. The coarse-grained clustering consists of 12 clusters, which are further subdivided into 53 clusters at the medium-grained level and 174 clusters at the fine-grained level. These plots illustrate the hierarchical clustering method that achieved the highest Silhouette scores, as detailed in Table 3 .

Figure 3 show the results for the coarse-grained, medium-grained, and fine-grained clustering.

To further detail the clustering results, various clusters from the three levels of clustering detail were examined in depth together with the domain experts from the LKR.NRW. Examples of the clusters identified are described below. Cluster labels are prefixed with 'c' (coarse-grained), 'm' (medium-grained), and 'f' (fine-grained) to indicate from which clustering granularity they originate.

##### Diagnosis-Only Clusters

At the coarsest level, the clustering results, shown in Fig. 1 , consist of 12 clusters. Even at this level of granularity, some treatment courses are clearly subdivided, allowing initial patterns to be identified. For example, clusters c11 and c6 consist of cases where only the diagnostic event is present, without any further treatment. The two clusters differ in age group (50–69 and >69, respectively). Patients under the age of 50 with no further treatment are found in cluster c2. However, this group forms its own cluster only at medium granularity and is still mixed with cases that have received additional treatments. At finer levels of granularity, these clusters are further subdivided into more detailed and nuanced clusters. For instance, cluster c11, which includes untreated cases between the ages of 50 and 69, is subdivided into two additional clusters based on differences in tumor grading.

##### Single-Treatment Clusters

Patients who have only undergone surgery are mostly found in clusters c9 and c8 at the coarse level. Cluster c9 includes cases with one surgery in the age group of 50 to 69, whereas cluster c8 consists of similar cases in the age group over 69. Cluster c1 mainly comprises cases with multiple surgeries but no further treatment. At the medium-grained level, cluster c8 is subdivided into three clusters: cluster m6 with BCS and a grading of 1 or 2, cluster m24 with mastectomy and a grading of 1 or 2, and cluster m18 with cases that received either a BCS or mastectomy with a grading of 3. In the fine-grained clustering, with 174 clusters, these three clusters are further subdivided. For example, cluster m18 is further subdivided by the type of surgery.

Similarly, clusters consisting of patients who received either only radiotherapy or only systemic therapy were found, although these patterns were identified only at medium and fine granularity.

##### Multimodal-Treatment Clusters

Clusters with multiple treatments, such as surgery followed by radiotherapy, are identified in clusters c7 and c10. Again, at finer levels of granularity, these clusters are further subdivided into more detailed and nuanced clusters. For instance, cluster c5 from the coarse-grained clustering result mostly consists of various groups of multi-treatment courses that are subdivided into five individual clusters in the medium-grained clustering ( Fig. 2 ): Cluster m42 consists of cases that underwent a BCS followed by hormone therapy. Cluster m5 consists of cases that underwent BCS followed by hormone therapy and additional radiotherapy. Cluster m38, which lies between cluster m5 and m42, contains cases with BCS followed by hormone therapy, and about half of the cases have additional radiotherapy. Cluster m38 differs from the previous two clusters primarily in terms of age group. Cluster m33 includes cases that underwent BCS followed by radiotherapy. Finally, cluster m25, which contains patients younger than 50 with only one surgery, stands out as it does not consist of multiple treatments. In the fine-grained clustering, these clusters are again further subdivided. For example, cluster m38 is subdivided into clusters f112 and f165, where treatment courses are differentiated based on the presence of additional radiotherapy.

##### A Closer Look at the Central Cluster

The largest cluster from the coarse-grained clustering, cluster c0, comprises 28% of all treatment courses. This cluster is highly heterogeneous and includes various combinations of surgery, radiotherapy, and systemic therapies, with most treatment courses consisting of three or more treatments. Chemotherapy is the most frequent therapy, occurring in 62% of treatment courses. Individual treatment patterns only become visible in the more fine-grained clustering results.

For instance, cluster m21 from the medium-granularity level predominantly comprises cases involving surgery, followed by chemotherapy, and, in some cases, also radiotherapy. At the next level of detail, this cluster is divided into three clusters: cluster f144 contains the cases with additional radiotherapy, cluster f50 contains cases with BCS, and cluster f11 mainly includes cases with mastectomy.

Cluster m47, another cluster within cluster c0, is split into two clusters (f136 and f137) at the fine-grained level. The treatment patterns in m47 are divided into two well-separated groups: radiotherapy followed by BCS (f137) and radiotherapy followed by BCS and hormone therapy (f136).

Cluster m17 also demonstrates that logical treatment groups sometimes only become visible at the finest granularity. In this cluster, we see a separation into surgery (95% mastectomy), followed by radiotherapy (f72), and radiotherapy only (f155). However, in f155 are entries for the temporal relationship to the primary surgery, suggesting that some surgery reports might be missing here.

**Fig. 4:**
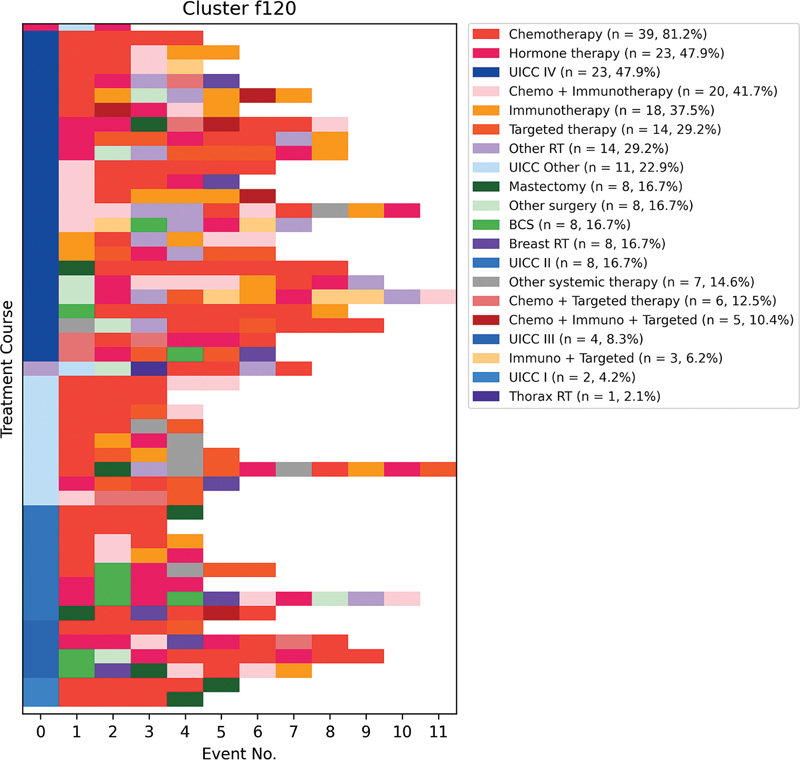
Sequence diagram of cluster f120 at the finest-grained level of clustering. Each row in the diagram represents a treatment course, and each cell in a row represents a treatment event, displayed by their most important attributes. This cluster remains less differentiated compared with others and is characterized by the use of multiple different systemic therapies, combined with surgeries and radiotherapies. The diagram highlights the common feature of combined treatment approaches within cluster f120. BCS, breast-conserving surgery; RT, radiotherapy.

An example of a cluster that remains less differentiated even at the finest-grained clustering is cluster f120. The common feature in this cluster appears to be the use of multiple different systemic therapies, combined with surgeries and radiotherapies ( Fig. 4 ).

### Survival Analysis

To further evaluate the content validity of the clustering results, a survival time analysis was performed based on the clustering procedure described in the previous sections. The objective of this analysis was to determine whether patients with similar prognostic tumor characteristics and similar ages form clusters that differ in terms of survival, even though survival time itself was not included in the cluster formation. Such an analysis has the potential to identify prognostically favorable (PF) or unfavorable (PU) treatment courses in the context of exploratory analyses and to subsequently investigate treatment paths that were associated with these courses of treatment.

#### Evaluation Setup

For this purpose, the total cohort of breast cancer patients diagnosed in 2019 was further restricted to patients aged 50 to 69 that survived at least 56 days after diagnosis. Additionally, patients with missing tumor grading were excluded due to the strong prognostic value of this characteristic. Within this refined group, two subgroups were formed:

1. PF cases with UICC stages I or II.
2. PU cases with UICC stages III or IV.

This stratification of patients was selected to minimize the likelihood of survival times differing between the clusters due to an accumulation of patients with particularly favorable or unfavorable prognostic tumor characteristics. Ideally, survival differences should result primarily from different treatment courses.

Suitable hyperparameters for clustering were selected for both cohorts, following a procedure similar to that described in section 3.1.1.1. The UMAP parameters were selected based on the visual inspection of the 2D images. For clustering, agglomerative hierarchical clustering, DBSCAN and HDBSCAN with different parameter configurations were used. Subsequently, the clustering results were restricted to the desired number of 15 to 30 clusters, and the result with the highest Silhouette score was selected. This process was performed separately for the PF and PU groups. The desired number of clusters of 15 to 30 was determined in consultation with subject matter experts, considering interpretability and a sufficiently high number of cases per cluster.

The parameter selection resulted in the following clustering results:

- PF clustering: UMAP n_neighbors 25, min_dist 0.1. Hierarchical clustering with, distance_threshold 4.7. 28 clusters with Silhouette Score 0.75
- PU clustering: UMAP n_neighbors 10, min_dist 0.01. DBScan with ε 0.4, *min_samples* 7. 19 clusters, 11 outliers with Silhouette Score 0.64

For a qualitative assessment of the clusters, the respective median treatment courses (medoid of the cluster) were used, which were previously prepared graphically.

The survival time of patients in the selected clusters was calculated using the Kaplan–Meier method and a landmark approach. 11 Overall survival was chosen as the endpoint. The start of survival was defined as the date of diagnosis + 56 days (landmark period). Right censoring was performed on the date of the last linkage of all tumor cases with the mortality data from the registry offices (June 30, 2023). Due to the choice of the diagnosis year 2019 and the censoring date, the 4-year survival was selected for the comparison of the clusters. Clusters in which no deaths occurred were excluded from the survival time analysis.

Based on the Kaplan–Meier analysis, a multiple Cox proportional hazards model was used to address residual confounding arising from differences in histologic type (lobular carcinoma vs. nonlobular carcinoma), age at diagnosis, grading, and UICC stage. The cluster with the highest observed 4-year survival in the Kaplan–Meier analysis was chosen as the reference category for cluster membership. Confounder-adjusted survival curves were then calculated using G-computation in order to graphically display the adjusted survival probabilities of the clusters. The adjustment was carried out using the parameters from the previously fitted Cox model.

To illustrate the treatment courses of clusters with particularly good or poor survival after adjustment for possible confounders, the cluster with the minimum, the median (in the case of an even number of clusters, the two median clusters), and the maximum hazard ratio (HR) were each described in more detail with regard to their treatment courses based on the Cox models.

The software package R was used for data preparation and all analyses. 12 The raw and adjusted survival curves were calculated with the *adjustedCurves* package. 13 The *survival* package was used to calculate the Cox proportional hazards models. 14

#### Results

The following section details the outcomes of the survival time analysis, highlighting the prognostic differences observed between the identified clusters. The results are presented for both PF and PU cases.

##### Prognostically Favorable Cases

After excluding one cluster without mortality events ( *n* = 138), the PF subgroup consisted of 4,830 patients with a mean age at diagnosis of 59.6 years (standard deviation [SD] = 5.9 years). The distribution of UICC stage in the PF subgroup was as follows: stage IA = 61.4%, stage IB = 2%, stage IIA = 27.1%, and stage IIB = 9.5%. The grading was distributed as 16.0% with G1, 59.0% with G2, and 25.0% with G3. The proportion of tumors of the lobular histological subtype was 12.6%.

Hierarchical clustering with a distance threshold parameter value of 4.7 was selected for clustering the PF group. The subgroup was thus divided into 28 clusters with an average size of *n* = 177 (SD = 140, range: 46–539).

**Fig. 5:**
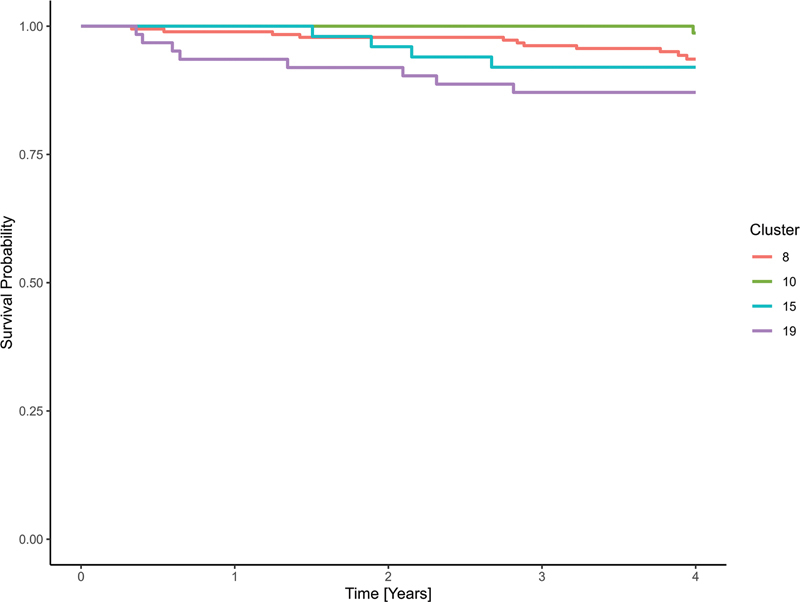
Raw Kaplan–Meier curves for selected clusters of the prognostically favorable subcohort, showing those with the lowest (cluster 10), highest (cluster 19), and median (clusters 8 and 15) hazard ratios.

**Fig. 6:**
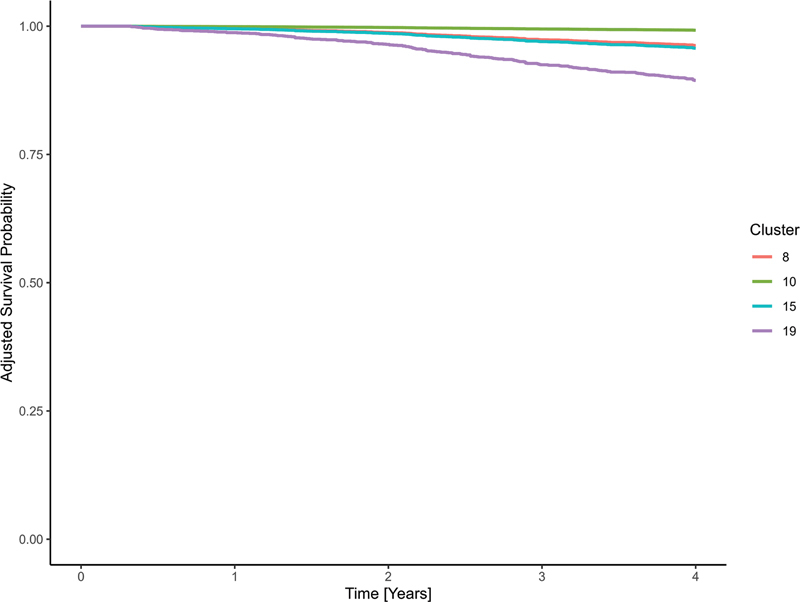
Kaplan–Meier curves for selected clusters of the prognostically favorable subcohort, based on a Cox proportional hazards model adjusted for age at diagnosis, gender, grading, histological subtype, and UICC stage. Displayed clusters represent the lowest (cluster 10), highest (cluster 19), and median (cluster 8 and 15) hazard ratios.

**Fig. 7:**
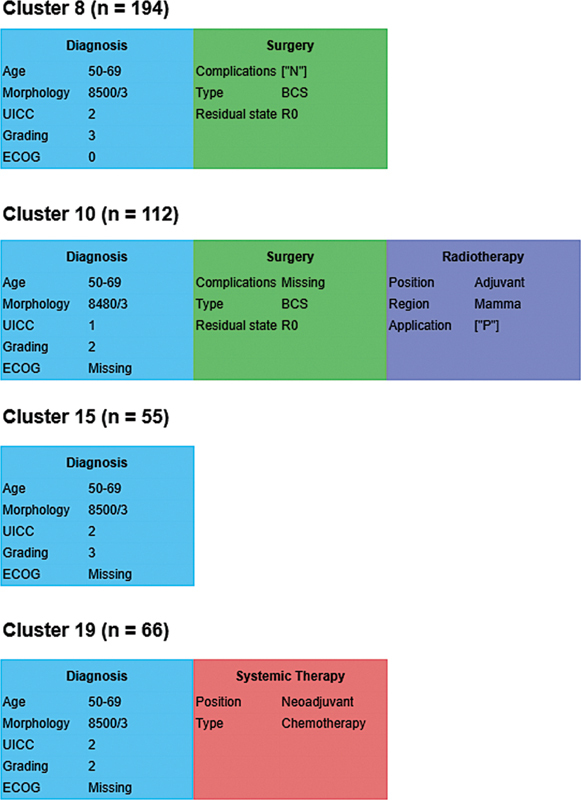
Medoid treatment courses for clusters 10, 19, 8, and 15 of the prognostically favorable subcohort. These clusters represent the lowest, highest, and median hazard ratios, respectively, and illustrate typical treatment patterns across the range of relative risk.

The crude 4-year survival probabilities of the individual clusters ranged from 87.1% (95% confidence interval [CI]: 0.79–0.96) to 99% (95% CI: 0.97–1.00). Figure 5 displays survival curves for the clusters with the lowest, highest, and median HRs, as estimated by the Cox model, to illustrate the range of prognostic variation while maintaining visual clarity. The results of the Cox model showed that even after adjustment for age at diagnosis, stage, grading, and histology, there were still differences in survival between the clusters ( Fig. 6 ). The HR for cluster membership ranged from 0.85 (95% CI: 0.05–13.72) in cluster 10 to 12.38 (95% CI: 1.52–100.10) in cluster 19 ( Table 4 ). The two median clusters were cluster 8 with an HR of 4.20 (95% CI: 0.53–33.37) and cluster 15 with an HR of 4.79 (95% CI: 0.52–44.24). The prognostically most favorable cluster 10 consisted predominantly of patients who had undergone BCS with adjuvant radiotherapy. The two median clusters in terms of HR consisted of patients for whom only BCS with R0 status and no complications were reported (cluster 8) and patients for whom no treatment information was available to the LKR.NRW (cluster 15). The least favorable prognosis cluster 19 included patients whose only available treatment was neoadjuvant chemotherapy. Figure 7 shows the medoid treatment courses for clusters with the lowest, highest, and median HRs, illustrating typical treatment patterns across the range of relative risk.

**Table 4:** Results of the Cox proportional hazards model of prognostically favorable breast cancer cases

	Hazard ratio	Standard error	95% CI lower bound	95% CI upper bound
Cluster 0	3.92	1.03	0.52	29.34
Cluster 1	3.52	1.23	0.31	39.63
Cluster 2	6.88	1.02	0.93	51.19
Cluster 3	3.98	1.06	0.5	31.49
Cluster 4	6.52	1.04	0.86	49.64
Cluster 5	3.68	1.08	0.44	30.69
Cluster 6	1.35	1.43	0.08	22.04
Cluster 7	8.82	1.06	1.1	70.78
Cluster 8	4.2	1.06	0.53	33.37
Cluster 9	2.89	1.06	0.36	23.19
Cluster 10	0.85	1.42	0.05	13.72
Cluster 11	4.79	1.05	0.62	37.2
Cluster 12	6.55	1.03	0.86	49.67
Cluster 13	8.11	1.06	1.02	64.42
Cluster 14	2.67	1.23	0.24	29.51
Cluster 15	4.79	1.13	0.52	44.24
Cluster 16	2.11	1.08	0.26	17.4
Cluster 17	3.16	1.23	0.28	35.29
Cluster 18	3.38	1.23	0.31	37.43
Cluster 19	12.38	1.07	1.52	101
Cluster 20	5.29	1.08	0.64	44.02
Cluster 21	6.4	1.16	0.66	61.77
Cluster 22	5.47	1.16	0.56	53.02
Cluster 23	5.76	1.07	0.71	46.91
Cluster 24	6.16	1.23	0.56	68.31
Cluster 26 (reference)	1	–	–	–
Cluster 27	2.87	1.16	0.3	27.72
Nonlobular Ca. (reference)	1	–	–	–
Lobular Ca.	1.89	0.27	1.12	3.19
UICC IA (reference)	1	–	–	–
UICC 1B	1.26	0.59	0.4	4
UICC 2A	1.54	0.18	1.07	2.21
UICC 2B	2.79	0.21	1.86	4.18
Grading G1 (reference)	1	–	–	–
Grading G2	0.81	0.29	0.46	1.44
Grading G3	1.25	0.32	0.67	2.31
Age at diagnosis	1.08	0.01	1.05	1.1

Abbreviation: CI, confidence interval.

##### Prognostically Unfavorable Cases

After excluding one cluster without death events ( *n* = 6), the PU group consisted of 618 patients with a mean age of 59.4 years (SD = 5.8 years). Of these, 20.0% were in UICC stage IIIA, 13.8% in stage IIIB, 14.9% in stage IIIC, and 51.3% in stage IV at diagnosis. In terms of grading, 3.4% were diagnosed with G1, 51.8% with G2, and 44.9% with G3.

The DBSCAN model with the parameters ε = 0.4 and *min_samples* = 7 was selected for the subgroup of PU cases. This identified 19 clusters, which had an average size of 32.8 patients (SD = 30.5, range: 6–123).

**Fig. 8:**
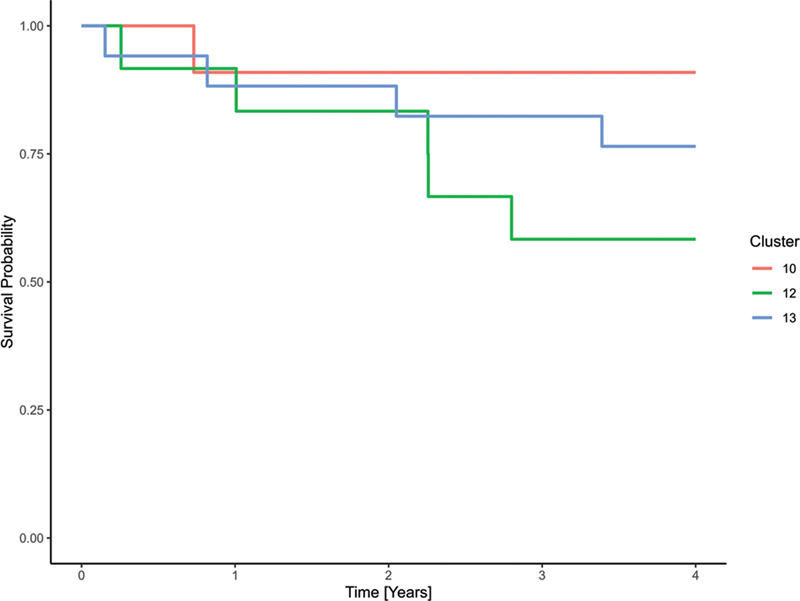
Raw Kaplan–Meier curves for selected clusters of the prognostically unfavorable subcohort, showing those with the lowest (cluster 10), highest (cluster 12), and median (cluster 13) hazard ratios.

**Fig. 9:**
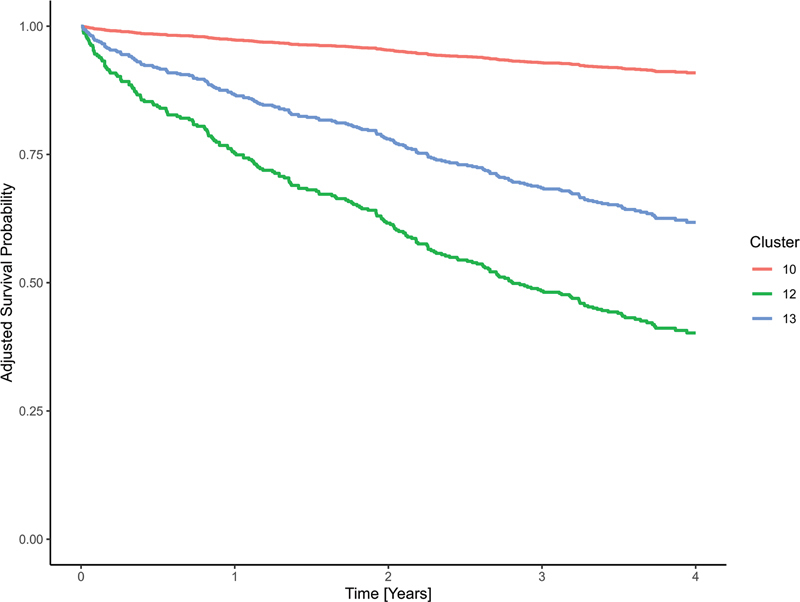
Kaplan–curves for selected clusters of the prognostically unfavorable subcohort, based on a Cox proportional hazards model adjusted for age at diagnosis, gender, grading, histological subtype, and UICC stage. Displayed clusters represent the lowest (cluster 10), highest (cluster 12), and median (cluster 13) hazard ratios.

The 4-year survival probabilities of the clusters ranged from 29.0% (95% CI: 16.7–50.39%) in cluster 8 to 90.9% (95% CI: 75.4–100.0%) in cluster 10. Figure 8 displays survival curves for the clusters with the lowest, highest, and median HRs. In the confounder-adjusted Cox model, HRs for cluster membership ranged from 1.0 in cluster 10 (the reference category of the cluster variable) to 11.00 [1.26–96.23] in cluster 12 ( Table 5 and Fig. 9 ). The median cluster in terms of adjusted HR was cluster 13 with an HR of 5.33 (95% CI: 0.59–48.41).

**Table 5:** Results of the Cox proportional hazards model of prognostically unfavorable breast cancer cases

	Hazard ratio	Standard error	95% CI lower bound	95% CI upper bound
Cluster 1 (outliers)	6.98	1.1	0.81	60.01
Cluster 0	9.53	1.03	1.26	72.18
Cluster 1	2.85	1.1	0.33	24.62
Cluster 2	2.63	1.03	0.35	19.7
Cluster 3	2.17	1.05	0.28	16.84
Cluster 4	2.62	1.06	0.33	21.01
Cluster 6	8.16	1.03	1.08	61.38
Cluster 7	5.32	1.02	0.72	39.02
Cluster 8	9.33	1.03	1.24	70.14
Cluster 9	1.5	1.42	0.09	24.22
Cluster 10 (reference)	1	–	–	–
Cluster 11	7.03	1.04	0.91	54.25
Cluster 12	11	1.11	1.26	96.23
Cluster 13	5.33	1.13	0.59	48.41
Cluster 14	3.54	1.13	0.39	32.4
Cluster 15	9.77	1.04	1.27	75.15
Cluster 16	6.79	1.04	0.89	51.64
Cluster 17	2.68	1.23	0.24	29.64
Nonlobular Ca. (reference)	1	–	–	–
Lobular Ca.	0.8	0.21	0.53	1.21
UICC 3A (reference)	1	–	–	–
UICC 3B	1.89	0.32	1	3.57
UICC 3C	1.22	0.35	0.61	2.43
UICC 4	2.69	0.29	1.52	4.76
Grading G1 (reference)	1	–	–	–
Grading G2	0.94	0.43	0.4	2.2
Grading G3	2.01	0.44	0.86	4.74
Age at diagnosis	1.02	0.01	0.99	1.04

Abbreviations: CI, confidence interval; UICC, Union for International Cancer Control.

**Fig. 10:**
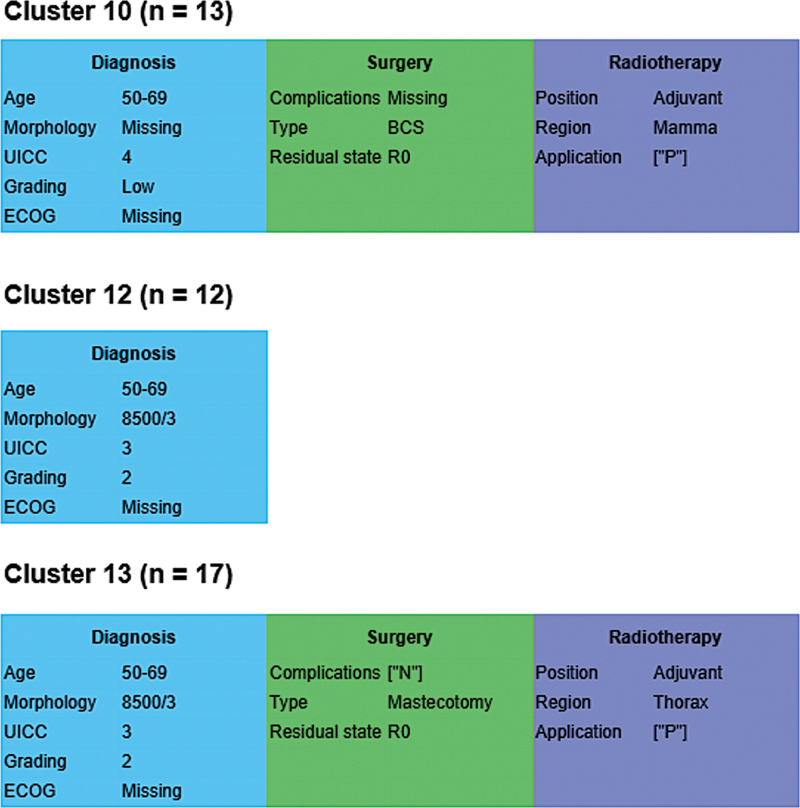
Medoid treatment courses for clusters 10, 12, and 13 of the prognostically unfavorable subcohort. These clusters represent the lowest, highest, and median hazard ratios, respectively, and illustrate typical treatment patterns across the range of relative risk.

Cluster 10 with the most favorable outcome, was characterized by BCS with R0 status and subsequent adjuvant radiotherapy. The typical patient in the cluster with the median HR (cluster 13) had undergone a mastectomy with R0 status and subsequent adjuvant radiotherapy. For patients in cluster 12 (highest HR), the LKR.NRW had no treatment information but only a diagnosis. Figure 10 shows the medoid treatment courses for clusters 10, 12, and 13.

## Discussion

### Discussion of the Clinical Relevance and Interpretability Results

As detailed in Section 3.1.2, our discussion with domain experts of the LKR.NRW showed that both the separation between clusters and the homogeneity within clusters were largely plausible. In the following, we summarize key findings from our discussions with domain experts.

We found that supplying varying levels of detail is important for the experts to explore and understand the results. As previously outlined, the coarse clustering with only 12 clusters, for instance, is often insufficiently granular to identify meaningfully distinct clusters. Depending on the use case and data of interest, results with different levels of detail may be necessary. The number of treatment events plays an important role in this regard, as treatment courses with fewer events see single attributes contributing more to the distance function than longer sequences. For instance, at medium granularity, treatment courses with only a single surgery event are already partially separated based on individual attributes, such as grading or type of surgery. In contrast, separation of longer sequences, like those in cluster c0 at the coarsest clustering result, is often only possible at the highest level of detail.

We found several clusters with treatment courses that appear clinically plausible. For example, treatment with BCS followed by radiotherapy in accordance with the treatment guidelines 15 can be found in several clusters, sometimes combined with other types of treatment, such as chemotherapy or hormone therapy treatment or further surgery. However, our clustering method was also able to find several clusters with unexpected treatment patterns in the data set that encourage further investigation. For example, cases where radiotherapy precedes BCS without subsequent radiotherapy, which does not align with the treatment guidelines. 15

Our clustering method also revealed several clusters with apparently implausible data. One notable example is the cluster of patients under the age of 50 without any apparent treatment. It is highly unlikely that patients under the age of 50 would not receive some form of treatment, especially since less than 10% of patients in the cluster reportedly have died. We assume that the corresponding treatment data were not transmitted to the cancer registry. Other examples include clusters with treatments before diagnosis, BCS after mastectomy, or adjuvant or neoadjuvant radiotherapy without corresponding surgeries. These cases demonstrate that clustering can make an important contribution to improving data quality by revealing previously unknown data problems.

The experts of the LKR.NRW made several suggestions as to how clustering of treatment courses could be applied in practice in the future. These suggestions included using clustering to uncover data quality problems by examining conspicuous or unexpected clusters more closely. Another idea was to present individual cluster results at the regularly organized quality conferences. This approach aims to raise interest in the respective topics and to discuss specific clusters with other groups of professionals (doctors, cancer registrars, etc.). Another potential approach is to utilize clustering as a preliminary selection process for identifying cohorts.

### Discussion of Survival Analysis Results

As presented in Section 3.2.2, we analyzed the survival time of the identified clusters after restricting the dataset to cases with similar tumor characteristics. This step aimed to reduce confounding and allow for a clearer comparison of treatment courses. Below, we discuss these findings in more detail.

The aim of the survival analysis in this work was not to draw conclusions about treatment efficacy but to explore whether treatment course-based clustering, performed independently of survival information, can identify subgroups of patients with different prognostic profiles. The analysis was therefore exploratory in nature and should be interpreted in the context of hypothesis generation, data quality assessment, and potential quality-of-care monitoring within cancer registries.

As expected, much of the observed variability in survival can be attributed to established prognostic factors such as UICC stage, tumor grading, histological subtype, and age at diagnosis. Nevertheless, differences between clusters persisted after adjustment for these variables, suggesting that patterns in treatment sequences, despite being derived without survival data, contain additional prognostic information. These residual differences should not be interpreted causally but instead indicate that treatment trajectories, as recorded in cancer registries, can reflect differences in care processes, data completeness, or patient selection.

In the PF group, cluster 10 with BCS followed by radiotherapy exhibited the most favorable adjusted survival. This aligns with current guideline recommendations and serves primarily as an internal validity signal for the clustering procedure. More relevant for the exploratory objective of this study were those clusters in which treatment deviated from expected patterns. One example is the PF cluster in which BCS was recorded without any subsequent radiotherapy. Since adjuvant radiotherapy after BCS is associated with lower local recurrence and improved survival, its apparent omission here may reflect either genuinely omitted radiotherapy or incomplete reporting to the registry. 16 17 Systematically identifying such clusters may support the development of registry-based quality indicators, for example, by monitoring whether radiotherapy is reported within a clinically plausible time frame following surgery.

Another noticeable finding in the PF group was cluster 19, in which neoadjuvant chemotherapy was the only documented treatment. This cluster showed the poorest survival in this subgroup. This does not allow conclusions regarding the effectiveness of neoadjuvant treatment but may suggest early mortality before definitive local therapy, refusal or contraindication of surgery, or missing surgery documentation. From a registry perspective, such patterns may be relevant for quality assurance, for instance by monitoring whether definitive local treatment is recorded within a specific period following neoadjuvant therapy. This is particularly pertinent given that guidelines still emphasize surgery following neoadjuvant systemic therapy in operable settings. 18

In the PU group, intercluster differences in survival were more pronounced, which is in line with the greater heterogeneity and overall poorer prognosis of this subgroup. Again, clusters with BCS and radiotherapy showed the most favorable outcomes. Of greater exploratory interest, however, were clusters in which only a diagnosis was recorded but no treatment information was available. These cases were associated with particularly poor survival and may represent patients with rapidly progressing disease, refusal or ineligibility for treatment, or incomplete reporting. For cancer registries, such clusters can be useful in identifying reporting delays, institutional differences in data submission, or specific care pathways (e.g., patients treated exclusively in outpatient settings). 19

Although the survival analysis does not permit conclusions about the effectiveness of specific treatments, it demonstrates that clustering can draw attention to treatment patterns associated with unexpected outcomes. These include BCS without subsequent radiotherapy, neoadjuvant-only therapy, diagnosis-only records, or unusually long gaps between treatment steps. Each of these patterns can be used to formulate testable hypotheses or support quality-related registry activities. Examples include:

- Whether radiotherapy is under-reported or omitted after BCS.
- Whether delays between neoadjuvant therapy and surgery vary between institutions and impact long-term outcomes.
- Whether diagnosis-only cases are overrepresented in specific reporting facilities, indicating issues in data completeness or care access.

These hypotheses lie outside the confirmatory scope of this study but illustrate how clustering can inform future research and support cancer registry workflows by identifying cases that may warrant follow-up, validation, or clinical discussion. This aligns with international recommendations that cancer registry data should be used not only descriptively but also to monitor completeness, timeliness, and plausibility of treatment documentation. 20

In summary, while the survival analysis confirms known prognostic relationships, its added value lies in showing that treatment-sequence clustering, without survival data as input, can identify groups with differing outcomes, including groups where outcomes deviate from expectations and where data completeness or care processes warrant attention. These findings support the use of clustering as an exploratory tool in cancer registries, particularly for generating hypotheses and highlighting potential targets for data quality improvement or quality-of-care evaluation.

## Conclusion and Outlook

We evaluated our approach on a dataset from the LKR.NRW consisting of 17,822 breast cancer cases diagnosed in 2019. First, we discussed the results with domain experts of the LKR.NRW and demonstrated the applicability of our approach. In a second evaluation step, we performed a survival time analysis of patients in an intercluster comparison and demonstrated that clinically relevant differences between clusters could be identified.

In most cases, the domain experts found the separation between clusters and the homogeneity within clusters to be plausible. We identified several clusters with clinically relevant treatment patterns, as well as clusters with unexpected treatment patterns that encourage further investigation. By examining conspicuous or unexpected clusters, we discovered several clusters indicating data quality problems. The experts are particularly interested in this use case for clustering. Furthermore, while it is important to note that the results of our survival time analysis alone do not allow any conclusions to be drawn about the effectiveness of the different treatment strategies found in the clusters, particularly because treatment information in cancer registries is often incomplete, our clustering method was able to find prognostically distinct groups of treatment courses. These findings highlight the potential of clustering to support exploratory analysis of treatment courses and survival time.

Future work will focus on refining and expanding our clustering approach in several directions: First, we plan to extend the method to include additional cancer diagnoses, allowing broader applicability across cancer registry data. Second, we aim to incorporate additional diagnostic events, such as the occurrence of metastases, to better capture the complexity of disease progression. Third, we will explore alternative distance measures, with the goal to further enhance clustering quality. Additionally, future work should focus on assessing the method's integration within cancer registry workflows.

## Footnotes

a https://github.com/lmcinnes/umap/issues/153 , Accessed October 24, 2025
